# A nomogram integrating machine learning-derived CT radiomics and clinical characteristics for prognostic assessment in patients with locally advanced esophageal squamous cell carcinoma treated with definitive chemoradiotherapy with or without immunotherapy

**DOI:** 10.1186/s12967-025-07387-1

**Published:** 2025-12-16

**Authors:** Mingxia Zhu, Lan Zhang, Chunxiang Cao, Jiao Xue, Huo Zhang, Xin Zhou, Songbing Qin

**Affiliations:** 1 Department of Radiation Oncology, The First Affiliated Hospital of Soochow University, Suzhou, 215006 China; 2 Department of Radiation Oncology, Tongren Hospital, Shanghai Jiaotong University School of Medicine, Shanghai, 200336 China; 3 Department of Radiation Oncology, Shanghai 10th People’s Hospital, Tongji University School of Medicine, Shanghai, 200072 China; 4 Department of Medical Oncology, Northern Jiangsu People’s Hospital, Affiliated to Yangzhou University/Clinical Medical College, Yangzhou University, Yangzhou, 225003 China; 5 Department of Oncology, First Affiliated Hospital of Nanjing Medical University, Nanjing, 210029 China; 6 Department of Oncology, The Affiliated Suqian First People’s Hospital of Nanjing Medical University, Suqian, 223812 China

**Keywords:** Radiomics, Machine learning, Esophageal squamous cell carcinoma, Chemoradiotherapy

## Abstract

**Objectives:**

This study aimed to build a radiomics signature using machine learning methods to estimate overall survival in patients with locally advanced esophageal squamous cell carcinoma (ESCC) who underwent definitive chemoradiotherapy (dCRT), and to verify its prognostic value across independent patient cohorts.

**Methods:**

We retrospectively included 200 ESCC patients with histological confirmation from three medical centers. Radiomics models were constructed employing machine learning algorithms. A predictive nomogram combining radiomics-derived risk metrics with clinical features was established. Model performance was assessed by the concordance index (C-index), time-dependent ROC curves, and decision curve analysis (DCA). Similar modeling approaches were also applied to an independent immunotherapy-treated cohort.

**Results:**

The developed radiomics signature exhibited modest predictive ability for overall survival in advanced ESCC patients treated with dCRT. High-risk individuals experienced reduced survival in the training cohort (*p* = 0.028) and validation cohort (*p* = 0.021) datasets, with similar findings observed in two external validation cohorts. The integrated nomogram combining clinical and radiomic features outperformed other predictive models and demonstrated potential clinical value for survival prediction. Within the immunotherapy-treated subgroup, the radiomics signature remained a statistically significant predictor of survival (*p* = 0.002), and the combined nomogram consistently exhibited acceptable prognostic performance.

**Conclusions:**

A reliable radiomics signature was established to effectively estimate survival outcomes in patients with advanced ESCC undergoing chemoradiotherapy or immunotherapy. Combining this model with clinical data enhanced its predictive capacity, underscoring its value for personalized prognostic evaluation.

**Supplementary Information:**

The online version contains supplementary material available at 10.1186/s12967-025-07387-1.

## Introduction

 Esophageal cancer ranks among the top ten globally in both cancer incidence and mortality [1]. China bears a disproportionate burden, accounting for more than 40% of global cases [2]. Among histological types, esophageal squamous cell carcinoma (ESCC) predominates, particularly within the Chinese population. Advances in cancer screening, early detection, and therapeutic strategies have contributed to recent declines in incidence and mortality. Nonetheless, early-stage esophageal cancer is often asymptomatic, and as a result, many patients are first identified only when the disease has already progressed to an advanced or metastatic stage [3]. For individuals with unresectable locally advanced disease, concurrent chemoradiotherapy (dCRT) remains the standard treatment [4]. However, outcomes remain poor, with disease progression observed in over half of individuals following dCRT. Reported progression-free survival (PFS) at three years ranges from 25% to 33%, and five-year overall survival (OS) rates remain below 25% [5].

Recent clinical trials have shown that combining chemoradiotherapy with immunotherapy may further enhance treatment efficacy. However, accurately predicting individual response to this combined modality remains a major clinical challenge [6, 7]. This is largely due to the lack of validated predictive biomarkers, the heterogeneity of tumor biology and immune responses among patients, and the limited ability of conventional imaging and staging systems to reflect dynamic treatment effects or identify early progression [8]. A reliable prognostic model could assist clinicians in stratifying patients by risk and implementing more personalized treatment strategies, particularly enabling early intensification of therapy in those at high risk of disease progression.

Current imaging-based staging systems provide limited prognostic information and fail to capture the underlying biological heterogeneity of esophageal cancer. Although several studies have identified molecular biomarkers capable of predicting treatment response to concurrent chemoradiotherapy or immunotherapy, these biomarkers generally require surgical specimens or invasive pathological biopsies [9–11].

Radiomics—a novel approach that extracts diverse, quantifiable data from conventional medical scans using high-throughput computational algorithms—is increasingly recognized as a valuable approach for enhancing prognostic evaluation [12, 13]. Radiomics models have shown potential in estimating treatment outcomes and overall survival among patients with esophageal carcinoma, thereby supporting clinical decision-making [14, 15]. However, clinical translation is still evolving. Existing studies are often exploratory in nature and show variability in methodology, imaging protocols, and feature extraction processes. Machine learning-based nomograms, in particular, have emerged as promising tools for integrating radiomic and clinical features to predict treatment outcomes across multiple cancer types and disease settings [16, 17]. Beyond oncology, similar predictive modeling approaches have also been successfully applied to nephrology and immunotherapy response prediction, underscoring the cross-domain utility of AI-assisted clinical tools [18, 19]. These developments highlight the importance of building standardized, interpretable, and reproducible models to improve generalizability and facilitate broader clinical adoption.

This study developed a radiomics signature derived from machine learning algorithms to estimate overall survival among individuals with locally advanced ESCC patients undergoing definitive chemoradiotherapy. Additionally, a comprehensive nomogram was built by integrating radiomic features and clinical prognostic variables. Notably, this model also demonstrated generalizability in patients treated with combined chemoradiotherapy and immunotherapy, suggesting its potential clinical utility across treatment modalities.

## Methods

### Study population

A cohort of 200 ESCC patients with histologically confirmed diagnoses who received standard curative treatment between January 2019 and December 2022 were retrospectively included. These cases were collected from three academic institutions: the First Affiliated Hospital of Soochow University (Center 1, *n* = 129), Shanghai Tenth People’s Hospital (Center 2, Cohort SH, *n* = 37), and Northern Jiangsu People’s Hospital Affiliated with Yangzhou University (Center 3, Cohort YZ, *n* = 34). Inclusion criteria included: (a) histopathologically confirmed ESCC diagnosis; (b) definitive chemoradiotherapy or its combination with immunotherapy; (c) availability of contrast-enhanced pre-treatment computed tomography (CT) scans; and (d) complete baseline clinical and follow-up data. Exclusion criteria included: (a) inadequate CT image quality or indistinguishable tumor lesions; (b) prior oncologic treatment; (c) Patients who discontinued PD-1 therapy after a single administration due to severe adverse events; (d) concurrent primary malignancies; (e) missing essential clinical data; or (f) lack of follow-up records.

All patients underwent intensity-modulated radiotherapy (IMRT) with prescribed doses ranging from 5000 to 6000 cGy. Concurrent chemotherapy primarily consisted of paclitaxel with platinum-based agents (cisplatin or carboplatin), whereas elderly or platinum-intolerant patients received oral tegafur-uracil (S-1). Additionally, 44 patients from center 1 were included in the immune checkpoint inhibitor (ICI) cohort and received PD-1 blockade alongside definitive chemoradiotherapy. Agents administered included camrelizumab, sintilimab, tislelizumab, or pembrolizumab, with the choice determined by the treating physician based on availability and clinical judgment. Patients received PD-1 inhibitors through different timing strategies. Specifically, 4 patients (9.1%) were treated with ICIs concurrently with CRT; 18 patients (40.9%) received ICIs sequentially after CRT; and 22 patients (50.0%) received induction immunotherapy followed by concurrent chemoradiotherapy and subsequent maintenance immunotherapy. Those who discontinued treatment after a single administration due to severe adverse events were excluded. Baseline demographic and clinical data of the patients are presented in Table 1.

**Table 1:** Characteristic of patients with ESCC in each cohort

Characteristic	Training Cohort (*n* = 51)	Testing Cohort (*n* = 34)	Cohort SH (*n* = 37)	Cohort YZ (*n* = 34)	ICI Cohort (*n* = 44)
Age Mean (SD)	69.04 (5.62)	68.76 (7.62)	70.65 (8.98)	69.53 (7.83)	66.05 (6.84)
Gender n (%)					
Female	11 (21.57)	7 (20.59)	7 (18.92)	10 (29.41)	8 (18.18)
Male	40 (78.43)	27 (79.41)	30(81.08)	24 (70.59)	36 (81.82)
Clinical Stage n (%)					
Ⅱ	4 (7.84)	5 (14.71)	12 (32.43)	7 (20.59)	14 (31.82)
Ⅲ	36 (70.59)	20 (58.82)	19 (51.35)	18 (52.94)	14 (31.82)
ⅣA	11 (21.57)	9 (26.47)	6 (16.22)	9 (26.47)	16 (36.36)
Immunotherapy modality					
Concurrent ICI + CRT n (%)					4(9.1)
Sequential ICI after CRT n (%)					18(40.9)
Induction + CRT + maintenance n (%)					22(50.0)
Hb Mean (SD) g/L	130.65 (14.29)	133.79 (13.92)			134.64 (14.07)
WBC Median (IQR)*10^9^/L	6.36 (5.27, 7.69)	6.36 (5.32,7.71)			6.63 (5.38, 8.24)
Neu Median (IQR) *10^9^/L	4.24 (3.18, 5.61)	4.33 (3.15,5.70)			4.34 (3.30, 6.05)
LY Median (IQR) *10^9^/L	1.45(1.28, 1.74)	1.68 (1.16,2.12)			1.45 (1.25, 1.79)
Mono Median (IQR) *10^9^/L	0.42(0.36, 0.52)	0.46 (0.33,0.58)			0.43 (0.33, 0.57)
PLT Median (IQR) *10^9^/L	205.00 (159.00, 249.50)	228.5 (162.0,268.0)			226.50 (193.00, 306.00)
NLR Median (IQR)	2.86 (2.09, 3.80)	2.54 (1.75, 4.25)			2.79 (2.26, 3.83)
PLR Median (IQR)	139.13 (100.00, 182.18)	139.66(102.99,172.58)			148.49 (119.02, 181.98)
LMR Median (IQR)	3.39(2.44,4.24)	3.69(2.57,5.06)			3.27 (2.78, 4.23)
SII Median (IQR)	585.03 (376.63, 853.27)	560.34(359.39, 888.06)			621.27 (453.37, 1,102.33)
CRP Median (IQR) g/L	5.63(1.83, 15.36)	4.87 (1.35, 13.83)			5.47 (1.03, 13.87)
Albumin Mean (SD) g/L	39.44 (3.88)	41.72 (4.28)			40.55 (3.73)
PNI Mean (SD)	46.99 (4.57)	49.97 (5.26)			48.41 (4.48)
Prealbumin Mean (SD) g/L	202.56 (46.11)	220.40 (63.34)			216.93 (53.82)

ESCC, esophageal squamous cell carcinoma; ICI, immune checkpoint inhibitor; CRT, chemoradiotherapy; Hb, hemoglobin; WBC, white blood cell count; Neu, neutrophil; LY, lymphocyte; Mono, monocyte; PLT, platelet; CRP, C-reactive protein; NLR, neutrophil-to-lymphocyte ratio; PLR, platelet-to-lymphocyte ratio; LMR, monocyte-to-lymphocyte ratio; SII, systemic immune-inflammation response index; PNI, prognostic nutritional index

**Fig. 1:**
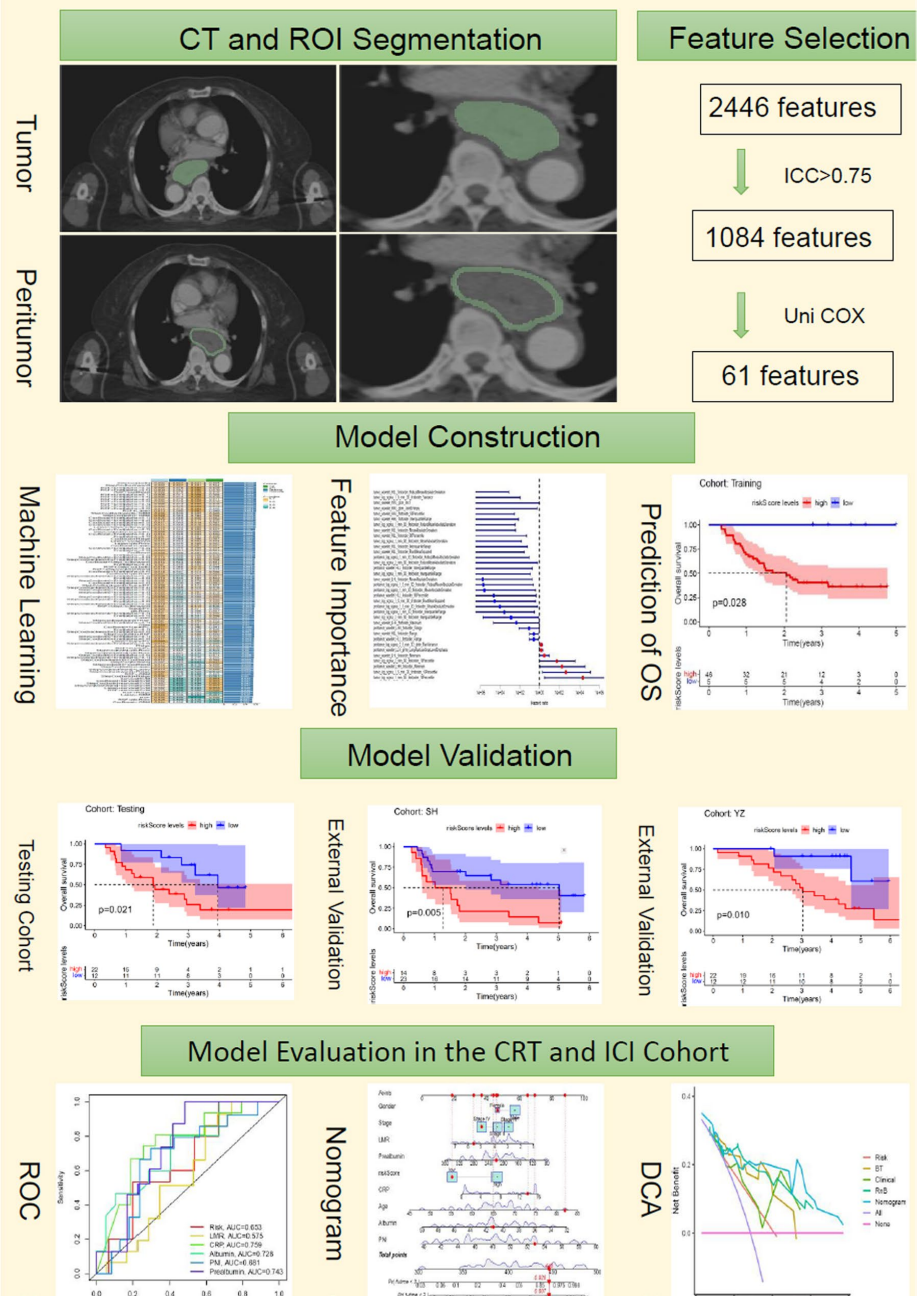
Workflow of our study. Contrast-enhanced CT images were manually segmented to delineate both tumor (intratumoral) and peritumoral ROIs. A total of 2,446 radiomic features were initially extracted. Reproducibility was evaluated using ICC, retaining 1,084 features with ICC > 0.75. Univariate Cox regression was used for further dimensionality reduction, resulting in 61 prognostic features. Machine learning algorithms were applied for model construction, including feature importance ranking and OS prediction. The model was trained in the CRT training cohort, evaluated in an internal testing cohort, and externally validated in two independent cohorts to assess its generalizability. The model was evaluated separately in the CRT and ICI cohorts, with assessment by ROC analysis, nomogram construction for individualized risk estimation, and DCA for clinical utility. Abbreviations: ROI, region of interest; ICC, intraclass correlation coefficient; OS, overall survival; CRT, chemoradiotherapy; ICI, immune checkpoint inhibitor; ROC, receiver operating characteristic; DCA, decision curve analysis

Patients were followed regularly until the last follow-up in December 30, 2023, with OS as the primary endpoint. Patients treated with definitive chemoradiotherapy at Center 1 (*n* = 85) were allocated randomly into two groups: a training set (*n* = 51) and a testing set (*n* = 34). Patients from Center 2 (*n* = 37) and Center 3 (*n* = 34) served as external validation cohorts. An additional independent validation dataset sourced from the Cancer Imaging Archive (TCIA; TCGA/TCIA-ESCA) included 12 qualified patients with baseline contrast-enhanced CT scans, alongside matched gene expression and clinical annotations from the TCGA project. Patient selection and study workflow are illustrated in Fig. 1 and Figure S1, respectively.

Ethical approval was obtained from the institutional review boards of the three participating centers, and the study was carried out in line with the Declaration of Helsinki. Given its retrospective design, the need for informed consent was waived.

### Data preprocessing

Contrast-enhanced CT images were anonymized and processed using 3D Slicer software (version 5.6.1; https://www.slicer.org/). Two senior radiation oncologists independently and blindly performed manual slice-by-slice tumor segmentation, without knowledge of clinical outcomes, to ensure unbiased delineation. Both the intratumoral (entire tumor area) and surrounding peritumoral regions were manually outlined as regions of interest (ROIs). The peritumoral ROI was defined as an annular shell with a 3-mm radial thickness, generated by 2 mm dilation and 1 mm erosion of the tumor boundary, based on previously reported peritumoral radiomics approaches [20]. All CT slices used for segmentation had a uniform thickness of 1 mm. Necrotic areas and adjacent non-tumoral structures such as vessels, airways, and bones were excluded. Feature extraction was conducted following the Image Biomarker Standardization Initiative (IBSI) using PyRadiomics [21].

CT images were acquired from three independent centers. All scans were performed using a tube voltage of 120 kVp, with tube currents ranging from 100 to 200 mAs and slice thicknesses between 3 and 5 mm, depending on the scanner and clinical protocol. To reduce variability in spatial resolution across scanners, all CT images were first resampled to a uniform voxel size of 1 × 1 × 1 mm³. To mitigate scanner-related variability, ComBat harmonization was applied to harmonize radiomic features across imaging devices. Next, z-score normalization was performed separately for the training, internal test, and external validation sets, using the mean and standard deviation calculated from the training set. Feature reproducibility was examined by calculating intra- and inter-observer intraclass correlation coefficients (ICCs) from 30 randomly selected cases, retaining only those with ICCs > 0.75 for subsequent modeling.

### Signature generation via multimethod machine learning

A multimethod machine learning framework comprising ten survival algorithms was applied as previously described [22, 23]. Initially, using univariate Cox analysis, 61 features showed significant associations with OS (*p* < 0.05). Subsequently, these features were further refined using a wrapper-based feature selection approach embedded within a leave-one-out cross-validation (LOOCV) framework. Specifically, 95 combinations of feature selection and survival modeling algorithms were constructed, including methods such as LASSO, ElasticNet (with multiple alpha values), RSF-based filtering, univariate Cox filtering, Stepwise Cox regression (forward, backward, and both directions), CoxBoost, RSF, and GBM. For each algorithm combination, feature selection and model training were performed exclusively within the training cohort. The resulting model was then applied—without refitting—to the internal validation cohort as well as two external validation cohorts (SH and YZ), and Harrell’s concordance index (C-index) was calculated separately for the training, internal validation, and external validation cohorts. The average C-index across the training and validation cohorts was used as an indicator of model robustness, following previously published multi-cohort model evaluation approaches [22, 23], without using the validation data for parameter tuning. The Stepwise Cox regression (both directions) algorithm, with p-value thresholds of 0.05 for inclusion and 0.10 for exclusion, achieved the highest average C-index among all candidates and was selected for final model construction, resulting in the selection of 35 most informative features. Since univariate Cox analysis was used solely for preliminary filtering rather than statistical inference, no multiple testing correction (e.g., FDR) was applied. The survminer package was employed to identify the optimal cut-off value within the training cohort. This training-derived threshold was applied unchanged to the internal testing and all external validation cohorts (SH, YZ, and ICI). For the combined single-center dCRT cohort, a cohort-specific cutoff was additionally computed for exploratory analysis, while the TCGA subset used a median split due to its small sample size.

### Clinical and radiomics-integrated nomogram development and validation

To evaluate the incremental prognostic value of the radiomics signature beyond conventional clinical parameters, we developed an integrated clinical–radiomics nomogram. A total of 22 candidate clinical variables were evaluated in this study. These included demographic characteristics (age and gender); tumor-related factors (clinical stage, Carbohydrate Antigen 19 − 9 [CA19-9], Carbohydrate Antigen 72 − 4 [CA72-4], carcinoembryonic antigen [CEA], and squamous cell carcinoma antigen [SCCA]); hematologic indicators (hemoglobin [Hb]; white blood cell [WBC], neutrophil, lymphocyte, monocyte, and platelet counts; albumin; prealbumin; and lactate dehydrogenase [LDH]); and inflammation/nutrition-related indices, including C-reactive protein (CRP), neutrophil-to-lymphocyte ratio (NLR), platelet-to-lymphocyte ratio (PLR), monocyte-to-lymphocyte ratio (LMR), systemic immune-inflammation index (SII), and prognostic nutritional index (PNI). We first applied univariate Cox regression to screen clinical features significantly related to OS (*p* < 0.05). A clinical-radiomics nomogram was subsequently developed by integrating the radiomics-derived risk score with clinical variables. Performance was assessed by C-index, time-dependent receiver operating characteristic (ROC) analyses, and decision curve analysis (DCA). For the ICI-treated cohort, the same model-building pipeline was applied. Clinical variables were re-evaluated using univariate Cox analysis, and the final integrated nomogram was reconstructed using cohort-specific radiomic and clinical data.

### Functional enrichment and immune landscape analysis

Patients in the TCGA/TCIA-ESCA cohort were stratified into low- and high-risk groups according to the median cutoff derived from the predefined risk score formula in the primary dataset. Because of the limited number of cases (*n* = 12), survival comparisons were not performed. Differentially expressed genes (DEGs) between risk groups were identified using the “limma” package in R, with |log₂ fold change| >1 and adjusted p-value < 0.05 as thresholds. GSEA (http://www.broadinstitute.org/gsea) was employed to explore GO and KEGG pathway enrichment among distinct risk groups. Significance was defined as NES > 1, NOM p-value < 0.05, and FDR q < 0.25. To explore links between the risk signature and the tumor immune microenvironment (i-TME), CIBERSORT was applied to estimate the distribution of 16 immune cell subtypes per sample. In parallel, hallmark and immune-related pathway activity was quantified using single-sample GSEA (ssGSEA).

### Statistical analysis

Normality testing determined variable distributions. Continuous variables were compared using either Student’s t-test or Mann–Whitney U test, while categorical comparisons were performed via chi-square or Fisher’s exact test. Survival outcomes were evaluated using Kaplan–Meier curves with significance tested via log-rank methods. All statistical procedures were conducted in R (version 4.4.1), with *p* < 0.05 (two-sided) considered significant. 

## Results

### Clinical characteristics

This study included 200 ESCC patients from three independent centers, consisting of 157 males and 43 females, with an average age of 69 years. Participants were assigned to five subgroups: 51 cases for training, and 34, 37, and 34 patients in the testing, SH, and YZ validation cohorts, respectively. Another 44 individuals receiving immunotherapy were grouped into the ICI treatment cohort. Most patients (*n* = 158) presented with stage III or IVA at diagnosis. By the end of follow-up, 110 patients had died, with a median overall survival of 35.28 months for the full cohort. Baseline characteristics, including peripheral blood cell counts (WBC, neutrophils, lymphocytes, monocytes, platelets), Hb, CRP, albumin, prealbumin, and composite indices (NLR, PLR, LMR, SII, PNI), are detailed in Table 1.

### Feature selection

A total of 1,223 radiomics features were extracted from a single ROI, including 14 shape features, 234 first-order features, 312 glcm features, 182 gldm features, 208 glrlm features, 208 glszm features, and 65 ngtdm features. Following test-retest analysis, 790 of the 1223 intratumoral features and 294 of the 1223 peritumoral features demonstrated satisfactory inter- and intra-observer ICCs (Figure S2). Subsequently, univariate Cox regression analysis identified 61 features significantly associated with OS in the training cohort. These selected features are detailed in Table S1.

### Radiomics signature construction

**Fig. 2:**
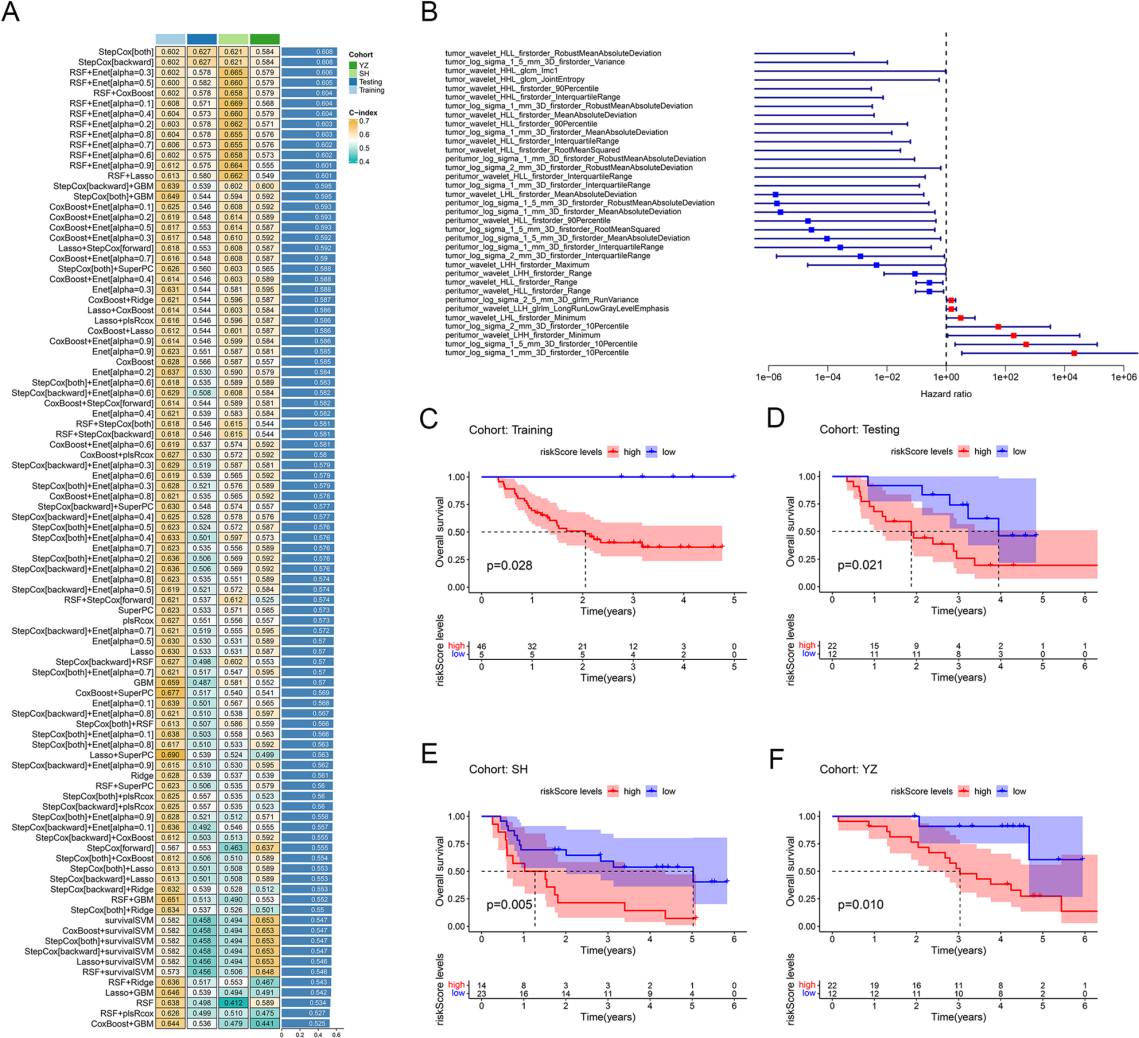
Radiomics signature construction and evaluation. **A**: A combination of 95 machine learning algorithms was generated. The C-index of each model was calculated through the Training, Testing, SH, and YZ cohorts and sorted by the average C-index. **B**: The univariate Cox regression analysis results of radiomics features in the training cohorts. (**C**–**F**) Survival analysis of ESCC patients with high risk score and low risk score in the training, testing, SH, and YZ cohorts. The median cutoff was determined in the training cohort and applied unchanged to all other cohorts

An ensemble framework was built using the 61 selected features to develop the radiomics signature. In the training cohort, a total of 95 machine learning pipelines combining various feature selection and survival modeling methods were tested to develop stable models. Among these, the Stepwise Cox regression (both directions) algorithm, using inclusion and exclusion p-value thresholds of 0.05 and 0.10, respectively, showed the best average C-index and was chosen for final model building (Fig. 2A). This approach identified 35 features as the most informative for prognostic modeling (Figs. 2B). Radiomic scores were subsequently computed for each patient across all cohorts. Patients were grouped into high- and low-risk categories based on the optimized cutoff value determined in the training cohort. Kaplan-Meier curves were generated to examine prognostic value, showing that high-risk patients had significantly reduced OS in the training cohort (*p* = 0.028, Fig. 2C). The same training-derived cutoff was then applied to the testing cohort, where high-risk patients similarly showed worse OS (*p* = 0.021, Fig. 2D). External validation in two additional cohorts (SH: *p* = 0.005; YZ: *p* = 0.01) further confirmed the generalizability and prognostic strength of the radiomics signature (Figs. 2E-F).

### Combined and clinical model construction and comparison in the dCRT cohort

**Fig. 3:**
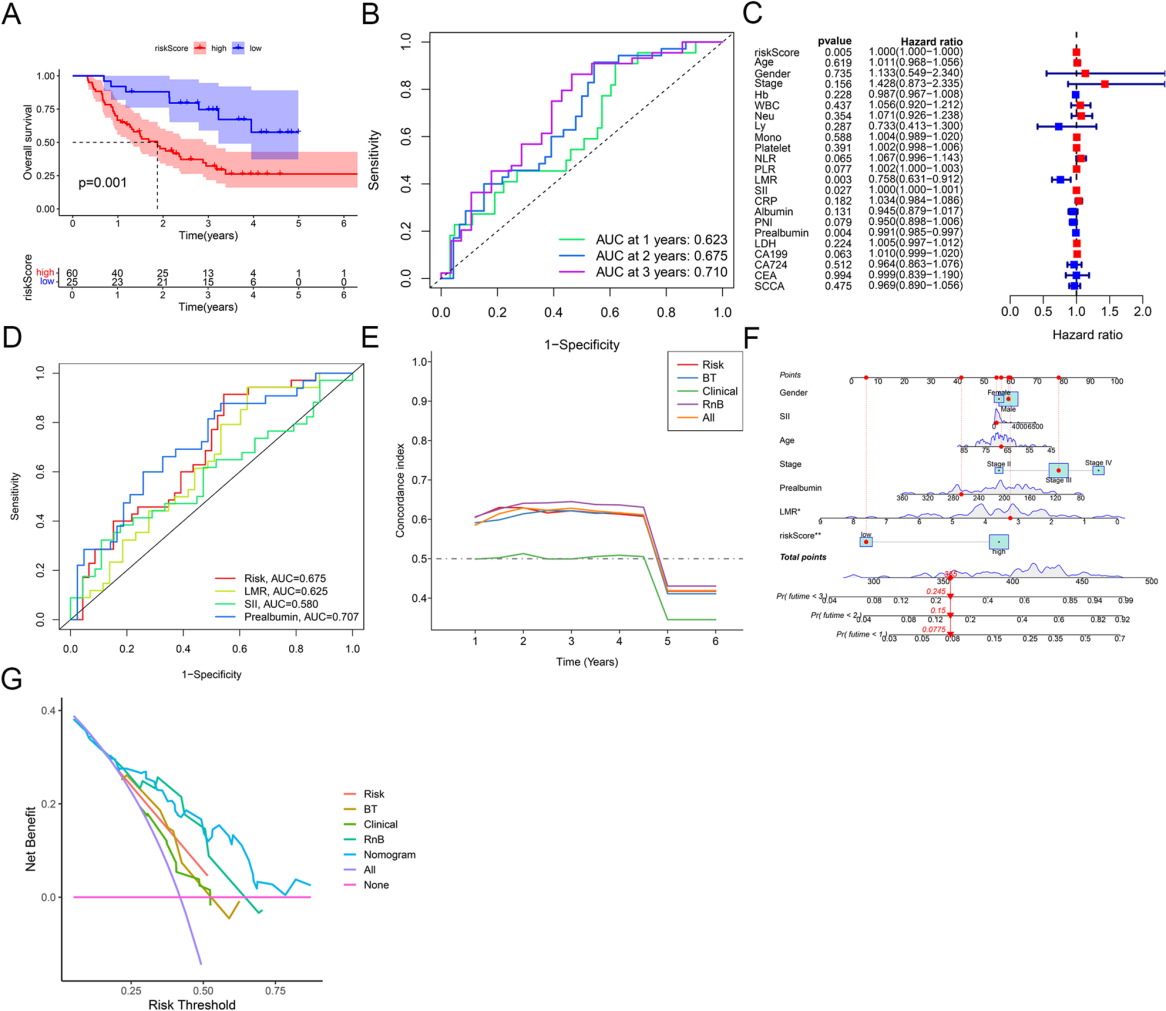
Construction and comparison of combined and clinical models in the definitive chemoradiotherapy cohort. **A**: Kaplan-Meier survival analysis of high-risk versus low-risk ESCC patients in the combined cohort (training and testing sets). **B**: ROC curves for 1-, 2-, and 3-year OS prediction using the optimized radiomics signature in the combined cohort. **C**: Univariate Cox regression analysis of candidate clinical variables in the combined cohort. **D**: ROC curves for 2-year OS prediction, including the radiomics risk score (Risk, red), lymphocyte-to-monocyte ratio (LMR, yellow), systemic immune-inflammation index (SII, green), and serum prealbumin level (Prealbumin, blue) in the combined cohort. **E**: Time-dependent C-index curves for prognostic models: radiomics risk score (Risk, red), blood test model (BT: LMR + SII + prealbumin, blue), clinical-only model (Clinical, green), combined model of risk score and blood test (RnB, purple), and all-features model (All, orange). **F**: Visual nomogram integrating the clinical-radiomics signature for individualized OS prediction. **G**: Decision curve analysis of the Risk, BT, Clinical, RnB, and All models for 2-year OS. ESCC, esophageal squamous cell carcinoma; ROC, receiver operating characteristic; OS, overall survival; LMR, lymphocyte-to-monocyte ratio; SII, systemic immune-inflammation index; BT, blood test (LMR + SII + prealbumin); RnB, risk score and blood test; All, all-features model

In the merged training and testing cohorts, Kaplan–Meier analysis with a recalculated cohort-specific cutoff showed significantly worse OS in high-risk patients (*p* = 0.001, Fig. 3A). ROC analysis showed the risk score’s AUCs were 0.623, 0.675, and 0.710 for 1-, 2-, and 3-year OS predictions (Fig. 3B). Univariate Cox analysis revealed that OS was significantly influenced by the risk score, LMR, SII, and prealbumin (Fig. 3C). These significant predictors were combined to build the clinical prognostic model. ROC curve analysis for 2-year OS prediction showed AUCs of 0.675 for the radiomics risk score (Risk), 0.625 for LMR, 0.580 for SII, and 0.707 for prealbumin, with prealbumin showing the highest discriminative ability (Fig. 3D). Based on these significant variables, we constructed multiple prognostic models: the radiomics-only model (Risk), the blood test model (BT: LMR + SII + prealbumin), the clinical-only model (Clinical), the combined model of risk score and blood test (RnB), and the all-features model (All). Time-dependent C-index curves comparing five prognostic models indicated that the combined model (RnB) outperformed the risk score, BT, and clinical variables in predicting OS (Fig. 3E). Using these findings, a clinical-radiomics nomogram was generated to forecast patient outcomes (Fig. 3F). DCA showed that the nomogram offered superior net clinical benefit across various threshold probabilities when predicting OS (Fig. 3G).

### Combined and clinical model construction and evaluation in the ICI cohort

**Fig. 4:**
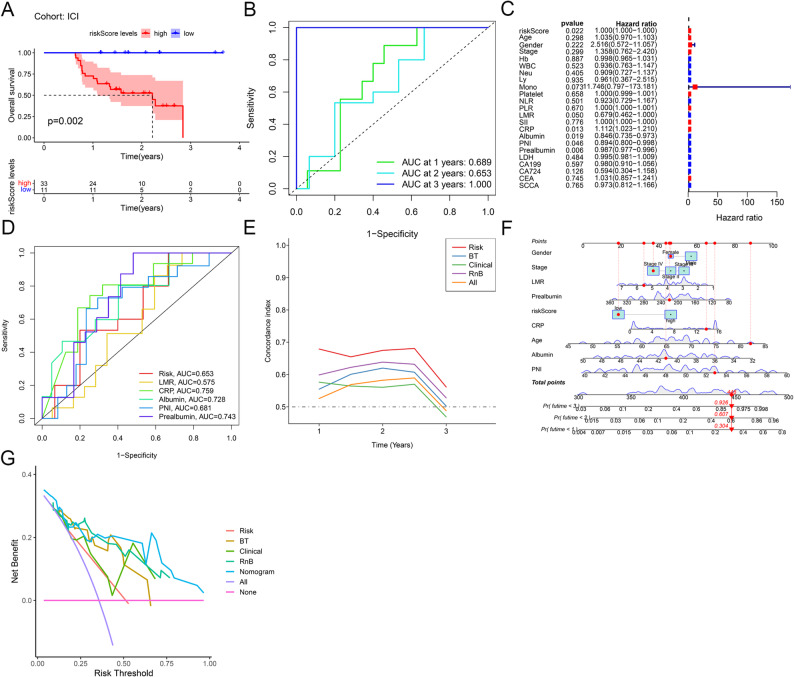
Construction and comparison of combined and clinical models in the ICI cohort. **A**: Kaplan-Meier survival analysis of high-risk versus low-risk ESCC patients in the ICI cohort. **B**: ROC curves for 1-, 2-, and 3-year OS prediction using the optimized radiomics signature in the ICI cohort. **C**: Univariate Cox regression analysis of candidate clinical variables in the ICI cohort. **D**: ROC curves for 2-year OS prediction in the ICI cohort, including the radiomics risk score (Risk, red), lymphocyte-to-monocyte ratio (LMR, yellow), C-reactive protein (CRP, green), albumin (Albumin, cyan), prognostic nutritional index (PNI, blue), and serum prealbumin level (Prealbumin, purple). **E**: Time-dependent C-index curves for prognostic models: radiomics risk score (Risk, red), blood test model (BT: LMR + CRP + albumin + PNI + prealbumin, blue), clinical-only model (Clinical, green), combined model of risk score and blood test (RnB, purple), and all-features model (All, orange). **F**: Visual nomogram integrating the clinical-radiomics signature for individualized OS prediction. **G**: Decision curve analysis of the Risk, BT, Clinical, RnB, and All models for 2-year OS. ESCC, esophageal squamous cell carcinoma; ROC, receiver operating characteristic; OS, overall survival; ICI, immune checkpoint inhibitor; LMR, lymphocyte-to-monocyte ratio; CRP, C-reactive protein; PNI, prognostic nutritional index; BT, blood test (LMR + CRP + albumin + PNI + prealbumin); RnB, Risk score and blood test; All, all-features model

In the ICI cohort, using the training-derived cutoff, Kaplan–Meier analysis demonstrated significantly shorter median OS in patients within the high-risk group compared to those in the low-risk group (*p* = 0.002, Fig. 4A). ROC analysis further supported the discriminative ability of the risk score, yielding AUC values of 0.689 and 0.653, and 1.000 for 1-, 2-, and 3-year OS, respectively (Fig. 4B). The perfect AUC observed at 3 years should be interpreted with caution, as only two patients in the ICI cohort survived beyond this time point, and both were correctly classified by the model. This result is likely attributable to the small sample size rather than a truly perfect predictive capability. Through univariate Cox regression analysis, significant prognostic variables identified for OS included risk score, LMR, CRP, albumin, PNI, and prealbumin (Fig. 4C). These significant variables were subsequently utilized to construct multiple prognostic models: the radiomics-only model (Risk), the blood test model (BT: LMR + CRP + albumin + PNI + prealbumin), the clinical-only model (Clinical), the combined model of risk score and blood test (RnB), and the all-features model (All). ROC curve analysis for 2-year OS prediction showed AUCs of 0.653 for Risk, 0.575 for LMR, 0.759 for CRP, 0.728 for albumin, 0.681 for PNI, and 0.743 for prealbumin, with CRP demonstrating the highest discriminative accuracy (Fig. 4D).

Time-dependent C-index curves comparing these five prognostic models revealed that the Risk model alone exhibited superior predictive performance relative to the other models (Fig. 4E). A clinical-radiomics nomogram was accordingly established to facilitate individualized prognostic predictions (Fig. 4F). Decision curve analysis confirmed the validity of the clinical-radiomics nomogram, demonstrating greater net clinical benefit than alternative models across a broad range of threshold probabilities (Fig. 4G).

### Functional enrichment and immune landscape analysis

**Fig. 5:**
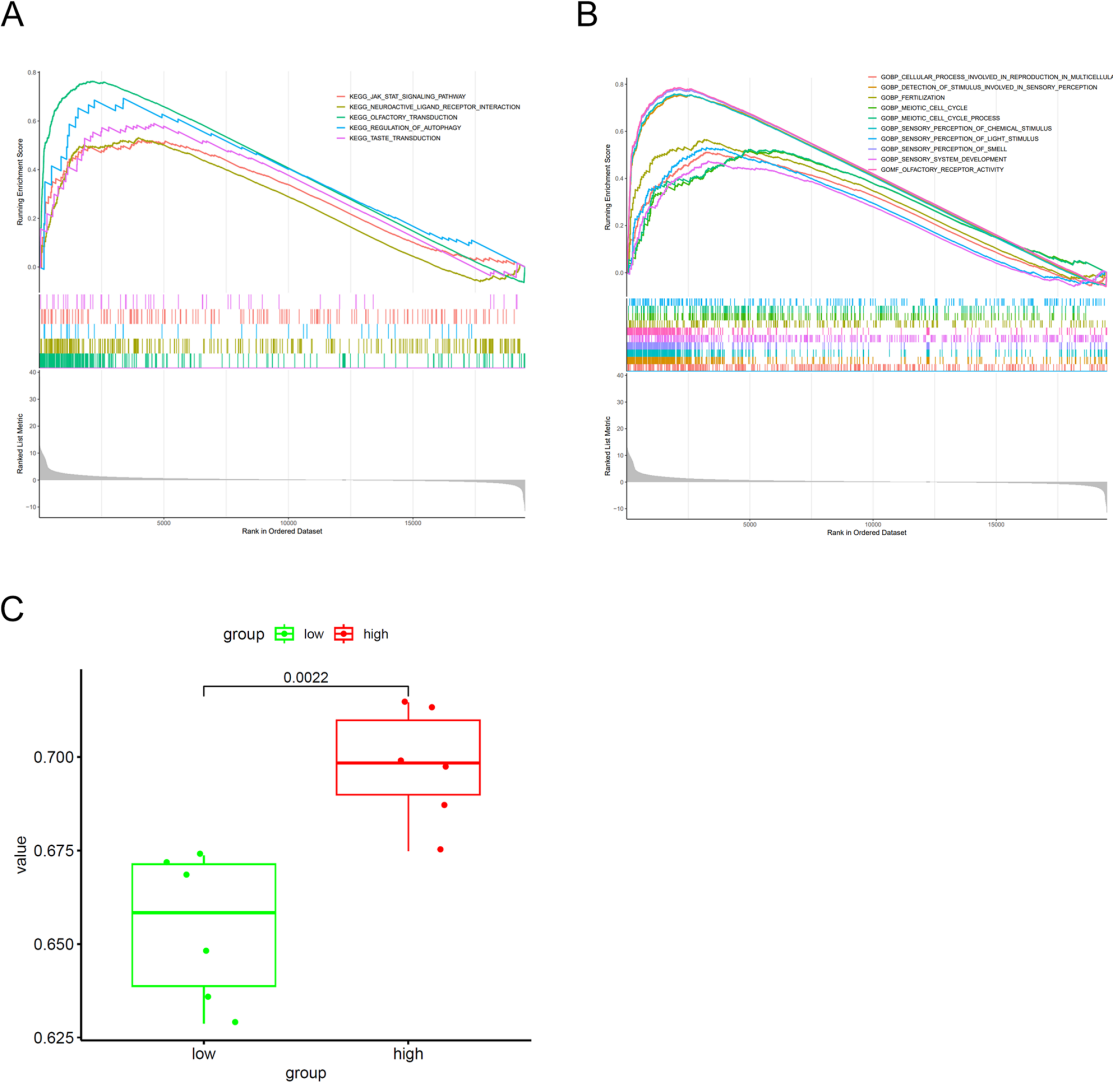
Functional enrichment analysis of risk-score–associated pathways in TCGA/TCIA-ESCA. **A**: Results of GSEA for risk score correlation with signaling pathways in KEGG collection. **B**: Results of GSEA for risk score correlation with signaling pathways in GO collection. **C**: ssGSEA-based pathway enrichment analysis demonstrating significant upregulation of the HALLMARK_MITOTIC_SPINDLE pathway in the high-risk group (*p* = 0.0022). Abbreviations: GSEA, gene set enrichment analysis; ssGSEA, single-sample GSEA; KEGG, Kyoto Encyclopedia of Genes and Genomes; GO, Gene Ontology

GSEA on 12 TCGA/TCIA-ESCA samples revealed marked pathway enrichment in the high-risk subgroup, which was defined by a median split owing to the limited sample size. Enriched pathways included JAK–STAT and autophagy-related signaling, both implicated in tumor progression and radioresistance (Fig. 5A). GO analysis further indicated enrichment of cell-cycle–related processes and sensory-perception–associated pathways in the high-risk group (Fig. 5B). Single-sample GSEA identified significant upregulation of the HALLMARK_MITOTIC_SPINDLE pathway in high-risk patients (*p* = 0.0022, Fig. 5C), which may be associated with adverse prognosis; however, this finding is exploratory and should be interpreted with caution given the limited sample size (*n* = 12).

However, CIBERSORT-based estimation of immune infiltration showed no notable differences in the proportions of 16 immune cell subsets across risk groups. Similarly, ssGSEA scores for 13 immune-related pathways showed no significant differences (Figure S3).

## Discussion

Prior studies have demonstrated that radiomics features extracted from pretreatment CT scans can reliably predict treatment outcomes in esophageal cancer patients receiving concurrent chemoradiotherapy [24–26]. Li et al. developed a 3D deep-learning radiomics model using pretreatment CT, which exhibited strong predictive ability for treatment response in thoracic ESCC [27]. Gong et al. conducted a multicenter study that built a hybrid nomogram integrating clinical factors, traditional radiomics, and deep-learning features from contrast-enhanced CT. This model effectively predicted local recurrence-free survival in ESCC patients treated with definitive chemoradiotherapy, outperforming single-modality approaches [28]. While Gong et al. highlighted the predictive benefit of combining deep-learning and radiomics features, our study focused on overall survival and extended the analysis to include patients receiving immunotherapy. Our model specifically incorporated radiomic features from both intra- and peritumoral regions, aiming to capture not only tumor-intrinsic heterogeneity but also microenvironmental characteristics. Most conventional radiomics research has emphasized intratumoral regions, under the assumption that they hold the most prognostic value, thereby overlooking the potential of peritumoral areas. Recent findings indicate that features from the peritumoral region can capture tumor heterogeneity and tumor microenvironment characteristics, potentially enhancing model performance. A recent study showed that combining intra- and peritumoral radiomics features improved Tumor-Node-Metastasis (TNM) staging prediction in resectable ESCC cases. Furthermore, radiomics-based TNM prediction was found to independently associate with RFS [20]. A dual-region radiomics model incorporating both intra- and peritumoral inputs achieved superior OS prediction compared to single-region models in proximal esophageal cancer post-chemoradiotherapy, underscoring the added prognostic value of peritumoral features [29]. In gastric cancer, radiomics features from both tumor and peritumoral regions were predictive of immunotherapy-related progression-free survival, suggesting their biological relevance in capturing immune patterns, including M1 macrophage infiltration [30]. Although our model incorporated both intra- and peritumoral features, we did not conduct a direct comparison with models using intratumoral features alone. Therefore, the incremental contribution of peritumoral features in our framework remains to be formally quantified. Future studies are warranted to assess the additive value of peritumoral information through head-to-head model comparison. Collectively, these findings underscore the necessity of integrating peritumoral radiomics features into predictive models, thereby capturing more comprehensive biological information and facilitating personalized clinical decision-making.

This study systematically evaluated multiple machine learning algorithms for developing a reliable prognostic model based on radiomic features. Among the tested algorithms, StepCox exhibited consistently strong performance in both training and external validation sets, as evidenced by its top-ranking C-index scores. Based on these comprehensive comparisons, the StepCox method was selected as the optimal modeling approach for integrating radiomics features to predict patient survival outcomes. The choice of StepCox was further justified by its methodological strengths, including greater interpretability, efficient feature selection, and stability in handling high-dimensional data, thereby enhancing the clinical applicability of the developed prognostic model [31].

Emerging evidence suggests that hematological markers representing systemic inflammation and nutritional balance have prognostic significance in esophageal cancer. Multiple studies have confirmed that reduced LMR is linked to unfavorable overall and disease-free survival, supporting its clinical utility as an accessible prognostic biomarker [32, 33]. Similarly, high SII values have shown an independent association with poor prognosis in ESCC patients receiving chemoradiotherapy, particularly when assessed alongside tumor-infiltrating lymphocyte levels, highlighting the interplay between inflammation and antitumor immunity [34]. Additionally, the CPR index—reflecting both nutritional and inflammatory status—has been shown to independently predict postoperative risks and long-term outcomes in ESCC patients treated surgically [35]. Consistent with previous literature, our univariate Cox analysis demonstrated that LMR, SII, and prealbumin were significant predictors of overall survival. Among these clinical variables, prealbumin exhibited the highest predictive accuracy, underscoring its critical role in assessing nutritional status and its impact on patient outcomes. Furthermore, our combined clinical-radiomics prognostic model demonstrated improved predictive performance compared to models based solely on clinical or radiomic features. These results highlight the potential value of incorporating multidimensional information—both systemic inflammatory/nutritional biomarkers and radiomics—for enhancing individualized prognostic assessment in patients with ESCC.

Although immunotherapy has revolutionized cancer treatment, the search for reliable biomarkers to predict treatment response continues to pose a clinical challenge [36]. In recent years, blood-based indicators have emerged as promising prognostic tools in esophageal cancer patients receiving immunotherapy. Retrospective studies indicated that baseline nutrition- and inflammation-related markers—such as high prealbumin, reduced CRP, and favorable indices such as PNI—were strongly linked to improved progression-free survival in advanced esophageal cancer patients receivingPD-1 blockade and chemotherapy [37]. Additionally, a combined SII-PNI score has demonstrated robust prognostic capability, with higher scores indicating worse survival outcomes among patients receiving immunotherapy [38]. Real-world evidence further supports the predictive role of baseline LMR and body mass index (BMI) in patients with metastatic EC receiving PD-1 inhibitor-based treatment, highlighting their potential utility in clinical practice [39]. In the present study, univariate Cox regression analysis demonstrated that hematological indicators such as LMR, CRP, albumin, PNI, and prealbumin significantly correlated with OS. Among these clinical variables, CRP demonstrated relatively higher discriminative accuracy, suggesting a potentially important role of systemic inflammation as a determinant of patient outcomes during immunotherapy.

CT-based radiomics signatures have emerged as promising tools for predicting immunotherapy outcomes across various cancer types, including lung and gastric cancers [30, 40]. Nevertheless, predictive models specifically tailored to evaluate immunotherapy responses in esophageal cancer patients remain scarce. Here, we developed a radiomics signature incorporating intratumoral and peritumoral features extracted from CT scans to estimate prognosis in ESCC patients receiving chemoradiotherapy. Notably, this radiomics signature demonstrated potential applicability in patients receiving combined chemoradiotherapy and immunotherapy, achieving an AUC of 0.653 for predicting 2-year OS related to immunotherapy. Moreover, a nomogram integrating the radiomics-derived risk score with clinical variables exhibited improved predictive performance compared to individual predictors alone, highlighting the potential additive value of this radiomics approach for personalized prognostic assessment.

Our GSEA analysis revealed significant enrichment of the JAK–STAT signaling pathway in the high-risk group, indicating its role in tumor progression and radioresistance. Previous studies have shown that JAK–STAT activation promotes proliferation and immune evasion in ESCC. For example, Pan et al. identified a confused cell identity (CCI) subtype of ESCC driven by TPM4–JAK/STAT–SOX2 signaling, linked to poor prognosis [41]. Moreover, carbon ion irradiation or LIF knockdown inhibited ESCC cell growth by suppressing JAK/STAT3 signaling, highlighting its role in radioresistance [42]. In addition, enrichment of cell cycle-related pathways, especially the HALLMARK_MITOTIC_SPINDLE gene set, suggests increased proliferation and genomic instability. This hallmark signature reflects mitotic activation and has been associated with poor prognosis and treatment response across cancers [43, 44]. However, these results were derived from a very small subset (*n* = 12) of TCGA/TCIA-ESCA patients, predominantly ESCC, and should be considered preliminary and hypothesis-generating. Larger, well-annotated transcriptomic datasets linked to imaging features will be essential to validate these associations and elucidate the underlying biological mechanisms.

Although our current analysis focused on survival prediction, accumulating evidence suggests that radiomic features may also serve as non-invasive biomarkers for tumor molecular characteristics, including immune-related markers such as PD-L1. Recent studies have demonstrated that radiomic signatures derived from CT scans are capable of predicting PD-L1 expression status across multiple cancer types. For instance, a multimodal deep learning model integrating radiomic, pathological, and clinical features achieved high predictive accuracy for PD-L1 levels and immunotherapy response in ESCC [45]. Similar findings were observed in bladder cancer [46] and non-small cell lung cancer [47], where radiomic features correlated with PD-L1 expression and immune response, underscoring the biological relevance of imaging phenotypes. However, due to the limited availability of molecular biomarker data in our cohort, we could not directly examine such associations. Future studies combining imaging and genomic data are warranted to enable radiogenomic correlation analyses and further enhance biological interpretability of radiomic models.

Despite the promising results, this study is subject to several important limitations. First, while the model achieved statistically significant prognostic discrimination, its predictive performance remains modest, underscoring the importance of continued refinement and external validation to support clinical translation. Second, the retrospective design and relatively small sample size—particularly the limited number of patients in the immunotherapy subgroup—may restrict statistical power and generalizability. Because the ICI cohort included only 44 patients, dividing the dataset into independent training and validation subsets would have severely compromised statistical power. Moreover, patients in this subgroup received different PD-1 inhibitors (camrelizumab, sintilimab, tislelizumab, or pembrolizumab) and treatment strategies (concurrent, sequential, or induction plus maintenance), introducing heterogeneity that could affect outcome interpretation. In addition, chemotherapy regimens varied within our cohorts, with some patients receiving platinum-based and others S-1–based therapy according to institutional protocols and physician discretion. While this diversity may introduce confounding effects, it also reflects real-world clinical practice and may enhance the model’s applicability across diverse treatment settings. Therefore, the findings from the ICI subgroup should be considered exploratory, and larger multicenter studies with more homogeneously treated ESCC cohorts are warranted to validate and refine the model. Furthermore, in the TCGA-ESCA cohort, ESCC cases were limited, as most samples represented adenocarcinoma, further constraining external validation. Third, the selection of cutoff values remains a limitation: while the training-derived cutoff was consistently applied across test and validation cohorts, exploratory recalibration in the combined dCRT and TCGA subsets highlights the need for robust threshold definition in larger, prospective datasets. Fourth, CT acquisition was performed across multiple centers using different scanners, introducing variability that was partially addressed through preprocessing and standardization before model building. Fifth, the manual segmentation process used was labor-intensive, underscoring the necessity for automated techniques in future research. Finally, incorporating multi-omics data and advanced imaging modalities like PET and MRI may further improve model performance.

## Conclusion

In summary, we developed a radiomics-based model using machine learning techniques that demonstrated the ability to predict overall survival in patients with locally advanced ESCC receiving chemoradiotherapy. The signature showed consistent performance and generalizability, with consistent prognostic performance validated in two independent external cohorts. Notably, it also retained predictive relevance in patients receiving combined chemoradiotherapy and immunotherapy. Furthermore, integrating the radiomics signature with key clinical variables into a comprehensive nomogram provided improved prognostic stratification compared to individual predictors. Our results highlight the potential application of radiomics-based models in clinical practice, although further prospective multicenter validation is warranted to confirm their clinical applicability.

## Supplementary Information

Below is the link to the electronic supplementary material.

Supplementary Material 1

## Data Availability

The data that support the findings of this study are available from the corresponding author upon reasonable request.

## Code availability

For machine learning model comparison, the implementation code was adapted from the publicly available source code of a previously published study (GitHub: https://github.com/Zaoqu-Liu/IRLS). The remaining custom scripts used for data preprocessing, feature extraction, model training (including parameter settings and model calibration), and performance evaluation have been uploaded to our GitHub repository (https://github.com/zhumingxia1/esophagus) for transparency and reproducibility.
