# Supplementary material for: A nomogram integrating machine learning-derived CT radiomics and clinical characteristics for prognostic assessment in patients with locally advanced esophageal squamous cell carcinoma treated with definitive chemoradiotherapy with or without immunotherapy
Source: J Transl Med. 2025 Dec 16;23:1398. doi: 10.1186/s12967-025-07387-1 (PMC12709767; doi:10.1186/s12967-025-07387-1)
Supplement: Supplementary file 1 — Supplementary Material 1 [file 12967_2025_7387_MOESM1_ESM.docx]

**Figure S1** Recruitment flowchart for the patients in this study. ESCC, esophageal squamous cell carcinoma; ICI, Immune Checkpoint Inhibitor.


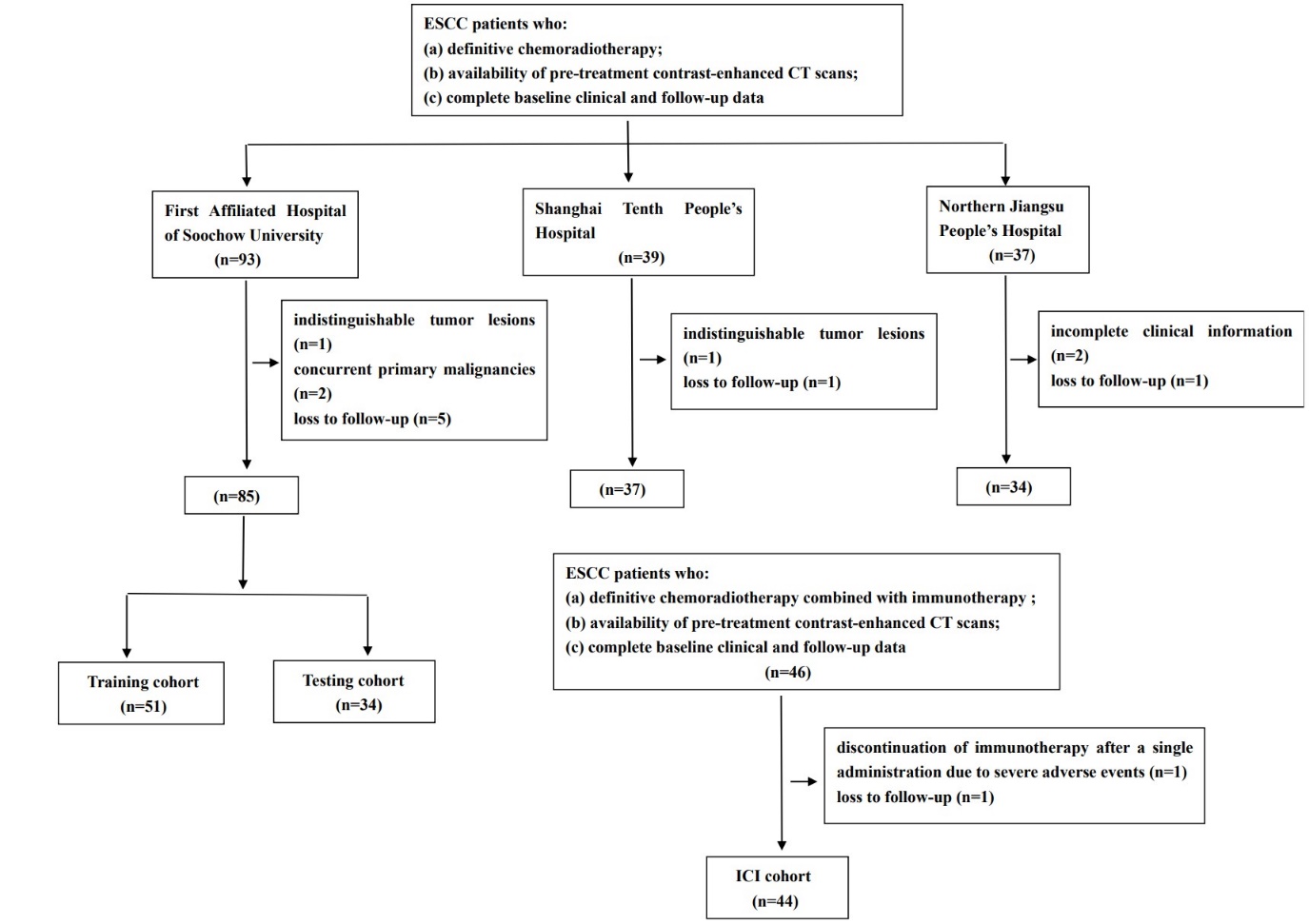


**Figure S2** ICC analysis for radiomics features in tumor and peritumoral regions**.** A: ICC values representing intra-observer variability within the tumor region features. B: ICC values representing intra-observer variability within the peritumoral region features. C: ICC values representing inter-observer variability within the tumor region features. D: ICC values representing inter-observer variability within the peritumoral region features. ICC, intraclass correlation coefficients.


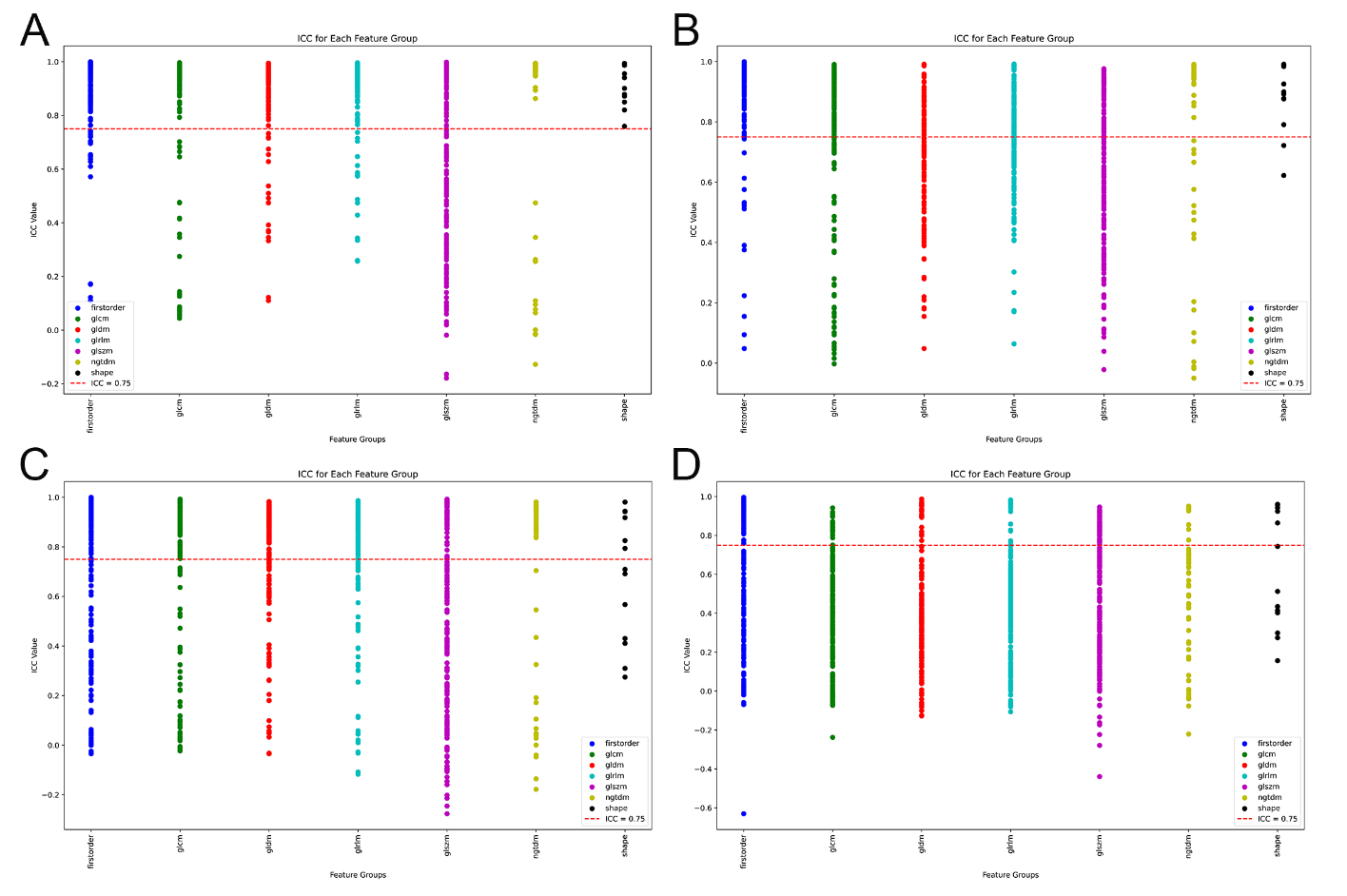


**Figure S3** Immune Landscape Analysis. A: CIBERSORT analysis of immune cell infiltration. B: ssGSEA-based pathway enrichment analysis of 13 immune-related pathways.


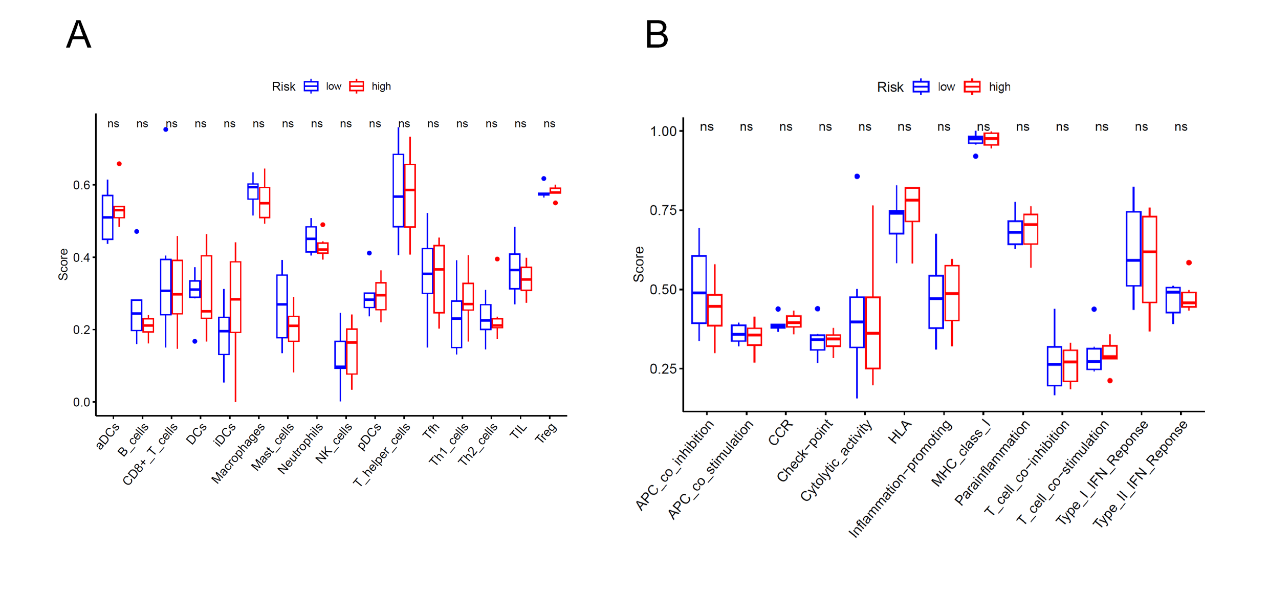


Table S1 Univariate Cox regression analysis of radiomics features associated with prognosis in ESCC

| radiomics features | HR | HR.95L | HR.95H | p value |
| --- | --- | --- | --- | --- |
| tumor_log-sigma-1-mm-3D_firstorder_10Percentile | 21084.48 | 3.350322 | 1.33E+08 | 0.02569 |
| tumor_wavelet-LHL_firstorder_10Percentile | 3229.291 | 5.269542 | 1978981 | 0.013606 |
| tumor_log-sigma-1-5-mm-3D_firstorder_10Percentile | 498.8136 | 1.984965 | 125349.8 | 0.027587 |
| tumor_wavelet-LLH_glcm_Imc2 | 247.4339 | 1.000447 | 61196.17 | 0.049981 |
| peritumor_wavelet-LHH_firstorder_Minimum | 190.1351 | 1.119472 | 32293.24 | 0.045173 |
| tumor_log-sigma-2-mm-3D_firstorder_10Percentile | 57.51806 | 1.002772 | 3299.181 | 0.049844 |
| tumor_wavelet-HLL_firstorder_Minimum | 34.56022 | 2.889345 | 413.384 | 0.005143 |
| peritumor_wavelet-HLL_firstorder_Minimum | 21.88942 | 2.106156 | 227.4982 | 0.009779 |
| tumor_wavelet-LHL_firstorder_Minimum | 3.087222 | 1.018322 | 9.359456 | 0.046366 |
| peritumor_wavelet-LLH_glrlm_LongRunLowGrayLevelEmphasis | 1.482949 | 1.004061 | 2.190243 | 0.047666 |
| peritumor_log-sigma-2-5-mm-3D_glrlm_RunVariance | 1.468194 | 1.042446 | 2.067823 | 0.027958 |
| peritumor_wavelet-LHL_ngtdm_Busyness | 1.00018 | 1.000029 | 1.000332 | 0.019359 |
| peritumor_wavelet-LHH_firstorder_Range | 0.087619 | 0.007915 | 0.969927 | 0.04716 |
| peritumor_wavelet-LHL_firstorder_Range | 0.560592 | 0.329071 | 0.955002 | 0.033225 |
| tumor_wavelet-HLL_firstorder_Maximum | 0.192636 | 0.038935 | 0.953086 | 0.043501 |
| tumor_wavelet-HHL_glcm_Imc1 | 1.45E-23 | 2.21E-46 | 0.947588 | 0.049766 |
| tumor_wavelet-LHH_firstorder_Maximum | 0.004406 | 2.08E-05 | 0.934911 | 0.047191 |
| tumor_wavelet-HHL_glcm_DifferenceEntropy | 1.24E-23 | 1.69E-46 | 0.916912 | 0.049624 |
| tumor_wavelet-LLH_glcm_Imc1 | 0.000822 | 7.47E-07 | 0.903265 | 0.046763 |
| tumor_log-sigma-2-mm-3D_firstorder_InterquartileRange | 0.001268 | 1.85E-06 | 0.868905 | 0.045269 |
| peritumor_wavelet-HLL_firstorder_Range | 0.272055 | 0.090906 | 0.814178 | 0.019937 |
| tumor_wavelet-HLL_firstorder_Range | 0.270816 | 0.095429 | 0.768548 | 0.014103 |
| tumor_log-sigma-2-mm-3D_firstorder_MeanAbsoluteDeviation | 9.11E-06 | 1.17E-10 | 0.710114 | 0.043432 |
| tumor_log-sigma-2-mm-3D_firstorder_RobustMeanAbsoluteDeviation | 8.64E-08 | 1.13E-14 | 0.658162 | 0.044251 |
| peritumor_log-sigma-1-5-mm-3D_firstorder_MeanAbsoluteDeviation | 9.35E-05 | 1.35E-08 | 0.648171 | 0.039777 |
| tumor_wavelet-HHL_glcm_JointEntropy | 1.49E-23 | 3.83E-46 | 0.576209 | 0.047621 |
| tumor_wavelet-LHL_firstorder_RobustMeanAbsoluteDeviation | 1.30E-08 | 3.78E-16 | 0.449829 | 0.040347 |
| peritumor_wavelet-HLL_firstorder_90Percentile | 2.14E-05 | 1.01E-09 | 0.449775 | 0.034237 |
| peritumor_log-sigma-1-5-mm-3D_firstorder_InterquartileRange | 0.003403 | 2.66E-05 | 0.436095 | 0.021726 |
| peritumor_log-sigma-1-mm-3D_firstorder_MeanAbsoluteDeviation | 2.51E-06 | 1.50E-11 | 0.418177 | 0.035548 |
| tumor_log-sigma-1-5-mm-3D_firstorder_RootMeanSquared | 2.81E-05 | 1.91E-09 | 0.412577 | 0.032291 |
| peritumor_log-sigma-1-mm-3D_firstorder_InterquartileRange | 0.000264 | 2.25E-07 | 0.310298 | 0.022344 |
| peritumor_log-sigma-1-5-mm-3D_firstorder_RobustMeanAbsoluteDeviation | 1.91E-06 | 1.42E-11 | 0.257643 | 0.028888 |
| peritumor_wavelet-HLL_firstorder_InterquartileRange | 2.12E-07 | 2.31E-13 | 0.194275 | 0.028244 |
| tumor_wavelet-LHL_firstorder_MeanAbsoluteDeviation | 1.72E-06 | 1.70E-11 | 0.173854 | 0.023976 |
| tumor_log-sigma-1-5-mm-3D_firstorder_InterquartileRange | 1.96E-05 | 2.30E-09 | 0.166746 | 0.018876 |
| tumor_wavelet-LHL_firstorder_RootMeanSquared | 4.91E-05 | 1.54E-08 | 0.15628 | 0.01591 |
| tumor_log-sigma-1-mm-3D_firstorder_RootMeanSquared | 1.06E-07 | 8.73E-14 | 0.129917 | 0.024744 |
| tumor_log-sigma-1-mm-3D_firstorder_InterquartileRange | 2.22E-07 | 3.97E-13 | 0.123524 | 0.023223 |
| peritumor_wavelet-HHL_firstorder_RootMeanSquared | 3.73E-16 | 1.56E-30 | 0.088881 | 0.035443 |
| peritumor_log-sigma-1-mm-3D_firstorder_RobustMeanAbsoluteDeviation | 3.65E-09 | 1.56E-16 | 0.085066 | 0.02479 |
| tumor_log-sigma-1-5-mm-3D_firstorder_MeanAbsoluteDeviation | 4.37E-08 | 2.61E-14 | 0.073233 | 0.020478 |
| peritumor_wavelet-HLL_firstorder_RootMeanSquared | 1.95E-07 | 5.29E-13 | 0.072291 | 0.018197 |
| tumor_wavelet-HLL_firstorder_InterquartileRange | 5.78E-11 | 5.44E-20 | 0.061494 | 0.02622 |
| tumor_wavelet-HLL_firstorder_90Percentile | 6.69E-12 | 9.11E-22 | 0.049067 | 0.026415 |
| peritumor_wavelet-HLL_firstorder_MeanAbsoluteDeviation | 4.37E-10 | 4.19E-18 | 0.04556 | 0.022146 |
| tumor_wavelet-HLL_firstorder_RootMeanSquared | 1.28E-09 | 5.81E-17 | 0.028347 | 0.017647 |
| peritumor_wavelet-HLL_firstorder_RobustMeanAbsoluteDeviation | 7.82E-16 | 2.36E-29 | 0.025961 | 0.028537 |
| tumor_wavelet-LHL_firstorder_Variance | 2.84E-14 | 4.34E-26 | 0.018563 | 0.02463 |
| tumor_log-sigma-1-5-mm-3D_firstorder_RobustMeanAbsoluteDeviation | 1.03E-11 | 6.09E-21 | 0.017521 | 0.019651 |
| peritumor_wavelet-HHL_firstorder_90Percentile | 9.34E-16 | 5.95E-29 | 0.014677 | 0.025597 |
| tumor_log-sigma-1-mm-3D_firstorder_MeanAbsoluteDeviation | 1.50E-11 | 1.55E-20 | 0.014477 | 0.018217 |
| peritumor_wavelet-HHL_firstorder_InterquartileRange | 6.19E-17 | 3.73E-31 | 0.010284 | 0.025487 |
| tumor_log-sigma-1-5-mm-3D_firstorder_Variance | 6.43E-25 | 4.02E-47 | 0.010281 | 0.032725 |
| tumor_wavelet-HHL_firstorder_InterquartileRange | 8.04E-19 | 8.75E-35 | 0.007386 | 0.026305 |
| tumor_wavelet-HLL_firstorder_MeanAbsoluteDeviation | 4.43E-15 | 5.40E-27 | 0.003636 | 0.018213 |
| tumor_log-sigma-1-mm-3D_firstorder_RobustMeanAbsoluteDeviation | 5.60E-17 | 9.87E-31 | 0.003172 | 0.020556 |
| tumor_wavelet-HHL_firstorder_90Percentile | 2.46E-19 | 2.06E-35 | 0.00293 | 0.023281 |
| peritumor_wavelet-HHL_firstorder_MeanAbsoluteDeviation | 5.84E-25 | 4.40E-46 | 0.000774 | 0.024535 |
| tumor_wavelet-HLL_firstorder_RobustMeanAbsoluteDeviation | 4.95E-25 | 3.18E-46 | 0.00077 | 0.02458 |
| tumor_wavelet-HHL_firstorder_RootMeanSquared | 5.43E-23 | 1.02E-41 | 0.000289 | 0.019788 |
